# Perioperative fluid administration and complications in emergency gastrointestinal surgery—an observational study

**DOI:** 10.1186/s13741-021-00235-y

**Published:** 2022-02-22

**Authors:** Anders W. Voldby, Anne A. Aaen, Roberto Loprete, Hassan A. Eskandarani, Anders W. Boolsen, Simon Jønck, Sarah Ekeloef, Jakob Burcharth, Lau C. Thygesen, Ann M. Møller, Birgitte Brandstrup

**Affiliations:** 1 Department of Surgery, Holbæk Hospital, part of Copenhagen University Hospitals, Smedelundsgade 60, 4300 Holbaek, Denmark; 2 Department of Anesthesiology and Intensive Care Medicine, Holbæk Hospital, Holbæk, Denmark; 3 Department of Surgery, Slagelse Hospital, Slagelse, Denmark; 4 Department of Emergency Medicine, Holbæk Hospital, Holbæk, Denmark; 5 Department of Surgery, Zealand University Hospital, Roskilde, Denmark; 6 Department of Population Health and Morbidity, University of Southern Denmark, Odense, Denmark; 7 Department of Anesthesiology and Intensive Care Medicine, Herlev Hospital, Herlev, Denmark; 8 Institute for Clinical Medicins, University of Copenhagen, Copenhagen, Denmark

**Keywords:** Fluid therapy, Intestinal obstruction, Intestinal perforation, Intraoperative care, Postoperative complications

## Abstract

**Background:**

The fluid balance associated with a better outcome following emergency surgery is unknown. The aim of this study was to explore the association of the perioperative fluid balance and postoperative complications during emergency gastrointestinal surgery.

**Methods:**

We retrospectively included patients undergoing emergency surgery for gastrointestinal obstruction or perforation. A perioperative fluid balance of 2.5 L divided the cohort in a conservative and liberal group. Outcome was Clavien-Dindo graded complications registered 90 days postoperatively. We used logistic regression adjusted for age, sex, American Society of Anesthesiologists’ classification, use of epidural analgesia, use of vasopressor, type of surgery, intraabdominal pathology, and hospital. Predicted risk of complications was demonstrated on a continuous scale of the fluid balance.

**Results:**

We included 342 patients operated between July 2014 and July 2015 from three centers. The perioperative fluid balance was 1.6 L IQR [1.0 to 2.0] in the conservative vs. 3.6 L IQR [3.0 to 5.3] in the liberal group. Odds ratio of overall 2.6 (95% CI 1.5 to 4.4), *p* < 0.001, and cardiopulmonary complications 3.2 (95% CI 1.9 to 5.7), *p* < 0.001, were increased in the liberal group. A perioperative fluid balance of 0–2 L was associated with minimal risk of cardiopulmonary complications compared to 1.5–3.5 L for renal complications.

**Conclusion:**

We found a perioperative fluid balance above 2.5 L to be associated with an increased risk of overall and cardiopulmonary complications following emergency surgery for gastrointestinal obstruction or perforation. A perioperative fluid balance of 0–2 L was associated with the lowest risk of cardiopulmonary complications and 1.5–3.5 L for renal complications.

**Supplementary Information:**

The online version contains supplementary material available at 10.1186/s13741-021-00235-y.

## Key points summary

- We aimed to study the effect of a perioperative fluid balance above 2.5 L on postoperative complications following emergency gastrointestinal surgery.
- We found that a perioperative fluid balance above 2.5 L was significantly associated with an increased risk of overall- and cardiopulmonary complications and that the predicted risk of cardiopulmonary complications was at a minimum at a perioperative fluid balance between 0 and 2 L compared to 1.5–3.5 L for renal complications.
- Our results, from this multicenter observational study, imply a clinical potential of an optimized perioperative fluid strategy in patients undergoing emergency gastrointestinal surgery.

## Introduction

Worldwide, more than 310 million patients undergo major surgery each year (Weiser et al., 2015). Mortality and complication rates are among the highest in patients undergoing emergency gastrointestinal surgery (Khuri et al., 2005; Tengberg et al., 2017). Perioperative intravenous fluid is given to replace fluid loss and to ensure the perfusion of the organs. However, escape to the extravascular space rapidly diminishes the circulatory effect. Interstitial edema may follow and counteract tissue oxygenation. Systemic sepsis and the trauma of surgery might further amplify the extravascular escape of intravenous fluids. Little is known about which fluid strategy that is associated with a better outcome during emergency gastrointestinal surgery.

Studies comparing a restrictive and a liberal fluid strategy in patients undergoing elective abdominal surgery have shown that a restrictive strategy reduces the risk of complications and length of hospital stay (Nisanevich et al., 2005; Abraham-Nordling et al., 2012; Lobo et al., 2002). Yet, a too restrictive fluid strategy may cause renal failure (Myles et al., 2017). A near zero-balance approach has been shown to reduce cardiopulmonary and tissue healing complications in elective abdominal surgery (Brandstrup et al., 2003). Based on these findings, programs of Enhanced Recovery After Surgery (ERAS) recommend a conservative perioperative fluid approach and a weight gain of no more than 2.5 kg (Feldheiser et al., 2016). Patients undergoing emergency gastrointestinal surgery may benefit from a similar restrictive perioperative fluid approach.

The pathophysiological differences between patients undergoing elective and emergency surgery are marked. Patients undergoing emergency surgery are usually older and have more co-morbidities, and postoperative complications and death are more frequent than in patients undergoing elective surgery (Ingraham et al., 2011; Becher et al., 2011). The perioperative fluid strategy is often challenged by preoperative deterioration of the patient. Periods with reduced fluid intake, excessive pathological fluid losses (e.g., vomiting), and a hyper-inflammatory state call for careful attention when administering intravenous fluids (Becher et al., 2012). Sepsis may accompany the condition and fluid administration is a key element in the treatment. However, the volume associated with a better outcome is uncertain, especially for the surgical patient with sepsis (Rivers et al., 2001; Mouncey et al., 2015a; Investigators et al., 2014a; Investigators et al., 2014b).

We hypothesized that a perioperative liberal fluid strategy increases the risk of complications following emergency surgery for gastrointestinal obstruction or perforation. The aim of this cohort study was to compare the association of a conservative and a liberal fluid balance with postoperative complications following emergency surgery for gastrointestinal obstruction or perforation, and subsequently study the influence of the perioperative fluid balance on each type of complication.

## Methods

Study approval was granted by the Danish Patient Safety Authority (3-3013-1999/1) and the Danish Data Protection Agency (REG-149-2016) prior to data extraction. Ethical approval for this study (J.nr. 16-000014) was provided by the Ethical Committee, Zealand Region, Denmark, on 14 December 2016. The requirement for written informed consent was waived by the committee. We retrospectively collected data on patients admitted between 1 July 2014 and 31 July 2015 at three teaching hospitals in the Region of Zealand, Denmark. The study sites offer treatment free of charge for a population of approximately 800,000 citizens. Local guidelines for intraoperative fluid administration during emergency gastrointestinal surgery were not present during the study period. The Strengthening the Reporting of Observational studies in Epidemiology (STROBE) statement was used in drafting this manuscript (von Elm et al., 2007).

We included all adult Danish residents undergoing emergency gastrointestinal surgery due to obstruction or perforation confirmed radiologically. Minor surgical procedures such as appendectomies, cholecystectomies, and endoscopic procedures were excluded. We defined emergency surgery as any intraabdominal procedure without planned delay. We excluded children (aged 17 years or younger), pregnant women, patients receiving regular dialysis, or patients with a traumatic or iatrogenic perforation. If eligible for inclusion, more than once patients were included only at the first procedure. We excluded patients who had had intraabdominal surgery 30 days prior to eligibility or patients without data on the intra- and postoperative fluid therapy. The Danish Civil Registration System provides uniform identification of every citizen through a personal identification number used to access all electronically stored medical and anesthetic records. It offers complete information on death for all Danish residents (Pedersen, 2011).

The primary exposure was the perioperative fluid balance starting from the induction of anesthesia and to the end of stay at the post-anesthetic care unit or the intensive care unit (ICU) for up to 24 h. Fluid administration included crystalloids, glucose-containing fluids, colloids, intravenous drugs, packed blood products, and per oral intake. Fluid loss included diuresis, aspiration, emptied ascites, blood loss, and perspiration calculated as 0.5 mL kg^−1^ h^−1^. The fluid balance was calculated as the difference between the fluid administration and the fluid loss. Patients were divided in a conservative and liberal group at a perioperative fluid balance of 2.5 L in alignment with the ERAS recommendations (Ljungqvist et al., 2017).

The primary outcome was complications until postoperative day 90. The Clavien-Dindo classification (CDC) (Dindo et al., 2004) graded the complications and they were grouped into overall, wound-related, cardiopulmonary, renal, or infectious. We omitted CDC grade 1 because we expected nearly all patients to have a grade 1 complication. A complication graded CDC ≥ 3 was defined as a major complication and required radiological, endoscopic, or surgical intervention or critical care, which we defined as an admission at the intensive care unit. Secondary outcome was major complications or death at postoperative day 90.

We registered the postoperative complications as follows: wound-related complications included superficial wound rupture, rupture of the fascia, or anastomotic leakage. Cardiopulmonary complications included cardiac arrhythmia, acute myocardial infarction, cardiac arrest, pleural effusion, pulmonary congestion, pulmonary edema, congestive heart failure, or respiratory failure (failure to wean > 48 h, requiring continuous positive airway pressure after the day of extubating, or re-intubation of any cause). Renal complications included the need for dialysis or other renal complications (nephritis or hydronephrosis treated with a nephrostomy catheter). Infectious complications included superficial wound infection, pneumonia, urinary tract infection, or cutaneous infection. A clinical doctor set the diagnosis and initiated medical treatment.

The three participating hospitals used identical software and uniform registration of variables. We screened the booking system for patients undergoing abdominal surgery. All emergency procedures meeting the inclusion criteria and unclassified cases were further explored. We accessed the medical and anesthetic records on each patient eligible for inclusion. The data collected preoperatively were physiological status, co-morbidities, sepsis-2 score, and American Society of Anesthesiologists’ (ASA) classification. Intraoperatively, we registered the fluid administration and loss as specified above, vasopressor use and dose, hypotensive episodes defined as mean arterial pressure < 50 mm Hg at any time intra- and postoperatively, and the use of epidural analgesia.

Case report forms were used for data collection by our medically trained team. All team members were trained in the use of the Clavien-Dindo classification. AAA and AWV collected the anesthetic data, fluid administration, and losses. Two independent team members assessed each patient file and registered data on complications in two separate case report forms. Regular audit by the project leader (AWV) corrected irregularities. The senior advisor (BB) was consulted in case of incongruity. Database entry was conducted twice and inconsistencies were corrected by revisiting the case report form.

### Statistics

Data were tested for normality and parametric or non-parametric statistics was used as appropriate. The primary outcome was analyzed with multiple logistic regression. Confounders included were settled between the authors and a statistician based on a priori knowledge of variables known to be associated with the fluid administration by the physician and the postoperative complications (Ford et al., 2007; Al-Temimi et al., 2012). We included sex, age, ASA class (grouped at I–II or III–V), use of epidural analgesia (yes or no), use of vasopressors (yes or no), the type of surgery performed (bowel resection, other procedure, or palliative surgery (exculpatory stoma formation or limited treatment)), the intraabdominal pathology (gastrointestinal obstruction or perforation), and the hospital (Holbæk, Slagelse, or Køge). Age was left skewed and the potency was used. In case of > 5% missing data of independent variables, multiple imputation was planned. We performed a subgroup analysis excluding patients with preoperative sepsis-2-score ≥ 3 or those admitted directly to the ICU after surgery. Additionally, we analyzed patients with major complications separately. The results are presented as odds ratio (OR) with 95% confidence interval (95% CI). Statistically significance was Bonferroni corrected based on five outcomes, thus defined by a two-sided *p*-value < 0.01. We presented the predicted risk of complications depending on the fluid balance on a continuous scale. A generalized additive model with smoothing splines and four degrees of freedom was used. The statistical plan was approved by the authors before commencing the analysis of data. The statistical software was R version 3.5.0 GUI 1.70 El Capitan©R, 2016 and RStudio version 1.1.453.

## Results

A total of 457 patients had emergency surgery with radiologically verified GI obstruction or perforation and were screened for inclusion. Of these, 342 patients were eligible for inclusion. Excluded were five patients because of pregnancy or age below 18 years, one had end-stage renal failure, 65 patients had GI surgery within 30 days before the index procedure, fifteen had an iatrogenic perforation, nine patients had already been included once, eleven patients had trauma surgery, two patients were of foreign nationality, and seven patients were missing fluid data from the perioperative period.

A perioperative fluid balance of 2.5 L divided the cohort in two groups of similar size (Table 1). More patients in the liberal group had a gastrointestinal perforation (54 (33%) vs. 30 (17%)). In agreement with this more patients in the liberal group had a preoperative sepsis score of 3–4 (36 (22%) vs. 15 (9%)) and an ASA score of III–V (86 (53%) vs. 69 (39%)) and were more frequently admitted to the ICU directly following surgery (53 (33%) vs. 15 (8%)).

**Table 1:** Baseline characteristics of the conservative or liberal fluid group of patients undergoing emergency gastrointestinal surgery

		Conservative group (perioperative balance ≤2.5 L), number of patients (%)	Liberal group (perioperative balance > 2.5 L), number of patients (%)
Number of patients		179	163
Sex	Female	100 (55.9)	93 (57.1)
Age group	Years (median (IQR) ^φ^)	70.0 [57.5, 79.0]	72.0 [66.0, 79.0]
Body mass index	Median (IQR)	23.9 [21.1, 26.8]	23.9 [21.5, 27.9]
	Missing	14	10
Smoking habits	Current smoker	55 (32.4)	55 (34.2)
	Missing	9	2
Alcohol intake, female/male	> 7/> 14 units week^−1^	15 (8.7)	24 (15.5)
	Missing	7	8
ASA classification	1–2	110 (61.5)	77 (47.2)
	3–5	69 (38.5)	86 (52.8)
Sepsis-2 score, preoperative	0–2	162 (91.5)	126 (77.8)
	3–4	15 (8.5)	36 (22.2)
	Missing	2	1
Co-morbidity^#^	Heart disease	45 (25.1)	39 (23.9)
	Hypertension	73 (40.8)	79 (48.5)
	Pulmonary disease	26 (14.5)	31 (19.0)
	Liver disease	10 (5.6)	5 (3.1)
	Renal disease	11 (6.1)	15 (9.2)
	Diabetes mellitus	19 (10.6)	29 (17.8)
	Active cancer disease	24 (13.4)	30 (18.4)
Diagnosis	Adhesions	94 (52.5)	61 (37.4)
	Crohn disease	3 (1.7)	2 (1.2)
	Diverticulitis	13 (7.3)	15 (9.2)
	Hernia, strangulated	7 (3.9)	7 (4.3)
	Intraabdominal cancer	23 (12.8)	30 (18.4)
	Perforated ulcer	12 (6.7)	15 (9.2)
	Arterial ischemia	4 (2.2)	5 (3.1)
	Volvulus	11 (6.1)	9 (5.5)
	Other*	12 (6.7)	19 (11.7)
Surgical indication	Gastrointestinal obstruction	149 (83.2)	109 (66.9)
	Gastrointestinal perforation	30 (16.8)	54 (33.1)
Surgical procedure	Bowel resection	59 (33.0)	98 (60.1)
	Other procedure^§^	102 (57.0)	49 (30.1)
	Palliative surgery^θ^	18 (10.1)	16 (9.8)
Laparoscopy		11 (6.1)	11 (6.7)
Primary anastomosis	Small bowel	16 (8.9)	21 (12.9)
	Ileo-colic	12 (6.7)	9 (5.5)
	Colo-colic	2 (1.1)	5 (3.1)
Time to surgery, hour			
From hospital admission	0–12 h	67 (37.4)	71 (43.6)
	> 12 h	111 (62.0)	92 (56.4)
	missing	1	0
From assessment by surgeon	Hour (median [IQR] ^φ^)	3.0 [2.0, 6.0]	3.0 [2.0, 6.0]
	Missing	1	0
Time of surgery, median [IQR]		1.6 [1.1, 2.3]	2.3 [1.6, 3.3]
		3	2
Time of anesthesia, median [IQR]		2.2 [1.8, 2.9]	3.0 [2.2, 4.0]
Immediate postoperative intensive care		15 (8.4)	53 (32.5)
Sepsis-2 score, postoperative	0–2	137 (76.5)	89 (54.6)
	3–4	38 (21.2)	72 (44.2)
	Missing	4	2

^#^Some patients have more than one co-morbidity. ^φ^Interquartile range. *Unclassified surgery on the small or large bowel. ^§^Adhesiolysis, gastro-duodenorrhaphia, herniotomy, or peritoneal lavage. ^θ^Exculpatory stoma formation or limited treatment

During surgery, the liberal group had more hypotensive episodes, yet patients receiving vasopressor treatment were comparable between the groups. Postoperatively, more patients had hypotensive episodes and received vasopressors in the liberal group (Table 2). The median [IQR] perioperative fluid balance was 1.6 L [IQR 1.0 to 2.0] in the conservative group and 3.6 L [3.0 to 5.3] in the liberal group (Table 2). The liberal group were given more fluid intra- and postoperatively; however, the fluid loss increased primarily due to increase in diuresis.

**Table 2:** Perioperative fluid administration, losses, and associated variables during and after emergency gastrointestinal surgery

	Conservative group (perioperative balance ≤2.5 L), median [IQR] or no. (%) *n* = 179	Liberal group (perioperative balance > 2.5 L), median [IQR] or no. (%) *n* = 163
*Intraoperative data*		
Fluid variables, mL		
iv^#^ crystalloids	1400 [950, 1830]	2360 [1600, 3280]
iv colloids	0 [0, 0]	0 [0, 500]
iv glucose containing fluids	0 [0, 0]	0 [0, 0]
iv blood products	0 [0, 0]	0 [0, 0]
iv other fluids	110 [60, 170]	190 [90, 280]
Total iv fluid administration	1610 [1120, 2040]	2750 [2090, 3750]
Total iv fluid administration (mL kg^−1^ h^−1^)	9.8 [7.5, 12.7]	13.3 [9.0, 18.2]
Missing, no.	3	0
Diuresis	120 [0, 380]	180 [70, 450]
Blood loss	0 [0, 130]	100 [0, 400]
Other loss	110 [70, 420]	120 [80, 260]
Total loss	490 [140, 1130]	600 [310, 1130]
Fluid balance	930 [570, 1290]	2030 [1550, 2790]
Hypotensive episodes	79 (44.1)	105 (64.4)
Vasopressor given	156 (87.2)	152 (93.3)
Ephedrine, mg, *n* = 118 / 100^§^	20.0 [10.0, 30.0]	17.5 [10.0, 30.0]
Norepinephrine, mg, *n* = 10 / 40^§^	1.5 [0.4, 3.4]	2.8 [1.8, 5.0]
Phenylephrine, mg, n = 94 / 112^§^	1.0 [0.4, 2.2]	2.8 [1.0, 5.7]
*Postoperative data*		
Fluid variables, mL		
iv crystalloids	720 [400, 1280]	1900 [1090, 3170]
iv colloids	0 [0, 0]	0 [0, 400]
iv glucose	0 [0, 0]	0 [0, 230]
iv blood products	0 [0, 0]	0 [0, 0]
iv other fluids	180 [5, 350]	410 [180, 1190]
Total iv fluid administration	950 [590, 1510]	2970 [1710, 5620]
Total iv fluid administration (mL kg^−1^ h^−1^)	3.5 [2.3, 4.8]	4.6 [3.7, 6.8]
Missing, no.	3	1
Diuresis	140 [0, 500]	530 [110, 1320]
Blood loss	0 [0, 0]	0 [0, 0]
Other loss	140 [80, 280]	340 [140, 770]
Total loss	270 [110, 830]	970 [270, 2240]
Fluid balance	520 [250, 850]	1750 [1110, 3110]
Hypotensive episodes	17 (9.5)	46 (28.4)
Missing, no.	0	1
Vasopressor given	22 (12.3)	71 (43.8)
Ephedrine, mg, *n* = 6 / 13^§^	15.0 [10.0, 20.0]	10.0 [10.0, 20.0]
Norepinephrine, mg, *n* = 12 / 47^§^	5.9 [3.4, 14.2]	12.8 [6.2, 20.0]
Phenylephrine, mg, *n* = 9 / 19^§^	2.2 [1.0, 8.1]	3.1 [0.5, 5.9]
*Perioperative fluid data*		
Epidural analgesia, no. (%)	77 (43.0)	70 (42.9)
Total iv fluid administration	2610 [2160, 3310]	6000 [4290, 8930]
Total iv fluid administration (mL kg^−1^ h^−1^)	5.9 [4.1, 7.8]	7.3 [5.4, 10.2]
Missing, no.	3	0
Total loss	920 [480, 2000]	1900 [960, 3350]
Fluid balance, mL	1580 [1000, 2040]	3620 [3020, 5340]
Fluid balance, mL kg^−1^ h^−1^	3.3 [1.7, 5.2]	4.7 [3.4, 7.2]
Missing, no.	3	0

^#^Intravenous. ^§^The result is presented for those who received vasopressor or inotropic as specified by the *n* = (conservative / liberal)

### Primary outcome

Altogether, 225 (65.8%) patients had complications. The overall risk of complications was significantly associated with the liberal fluid group with an adjusted OR of 2.6 (95% CI 1.5 to 4.4), *p* < 0.001 (Table 3). No data were missing of the independent variables in the regression model. Subgroup analysis revealed a significantly increased risk of cardiopulmonary complications, OR: 3.2 (95% CI 1.9 to 5.7), *p* < 0.001 in the liberal group.

**Table 3:** Logistic regression analysis on the association between the perioperative fluid balance and postoperative complications following emergency gastrointestinal surgery

Complication	Conservative group *N* = 179 No. of patients (%)	Liberal group *N* = 163 No. of patients (%)	Crude		Adjusted analysis^*¤*^	
			OR (95% CI) *	*p*	OR (95% CI) *	*p*
Primary outcome						
Overall complications	98 (58.0)	127 (73.4)	2.9 (1.8–4.7)	< 0.001	2.6 (1.5–4.4)	< 0.001
Subgroups of outcome						
Wound-related	39 (23.1)	48 (27.7)	1.5 (0.9–2.5)	0.105	1.6 (0.9–2.7)	0.123
Superficial wound rupture	18	25				
Rupture of the fascia	20	20				
Leakage of the anastomosis	1	3				
Cardiopulmonary	45 (26.6)	89 (51.4)	3.6 (2.3–5.7)	< 0.001	3.2 (1.9–5.7)	< 0.001
Arrhythmia	14	28				
Acute myocardial infarction	2	2				
Cardiac arrest	2	0				
Pleural effusion	9	17				
Pulmonary congestion	5	14				
Pulmonary edema	2	2				
Respiratory failure	11	26				
Renal	7 (4.1)	15 (8.7)	2.5 (1.0–6.7)	0.053	-	-
Need for dialysis	2	3				
Other renal^§^	5	12				
Infectious	73 (43.2)	90 (52.0)	1.8 (1.2–2.8)	0.008	1.6 (1.0–2.5)	0.071
Wound infection	14	12				
Pneumonia	35	65				
Urinary tract infection	18	11				
Other infections	6	2				
	**Major complications**					
Secondary outcome						
Major complication	46 (27.2)	65 (37.6)	1.9 (1.2–3.0)	0.005	1.6 (1.0–2.7)	0.077
Subgroups of outcome						
Wound-related	23 (13.6)	27 (15.6)	1.3 (0.7–2.5)	0.333	1.2 (0.6–2.4)	0.606
Superficial wound rupture	3	4				
Rupture of the fascia	19	20				
Leakage of the anastomosis	1	3				
Cardiopulmonary	22 (13.0)	45 (26.0)	2.7 (1.6–4.9)	0.000	2.5 (1.3–4.9)	0.006
Arrhythmia	1	3				
Acute myocardial infarction	4	2				
Cardiac arrest	2	2				
Pleural effusion	3	9				
Pulmonary congestion	0	0				
Pulmonary edema	2	4				
Respiratory failure	10	25				
Renal	5 (3.0)	12 (6.9)	2.8 (1.0–8.9)	0.061	-	-
Need for dialysis	2	3				
Other renal	3	9				
Infectious	14 (8.3)	15 (8.7)	1.2 (0.6–2.6)	0.647	1.1 (0.5–2.5)	0.874
Wound infection	10	3				
Pneumonia	4	12				
Urinary tract infection	0	0				
Other infections	0	0				
Death at postoperative day 90	36 (21.3)	51 (29.5)	1.8 (1.1–3.0)	0.019	1.3 (0.7–2.4)	0.477

^¤^Clinical risk factors adjusted for in the model: sex, age in the potency, ASA class (dichotomized at ASA class 3), use of epidural analgesia (yes or no), use of vasopressors (yes or no), the type of surgery (bowel resection, palliative surgery, or other procedures), gastrointestinal obstruction or perforation, and the Hospital (Holbæk, Slagelse, or Køge). *OR odds ratio, *95% CI* 95% confidence interval. ^§^Hydronephrosis with nephrostomy catheter or treatment stalled due to renal failure. A *p*-value < 0.01 is considered significant

**Fig. 1:**
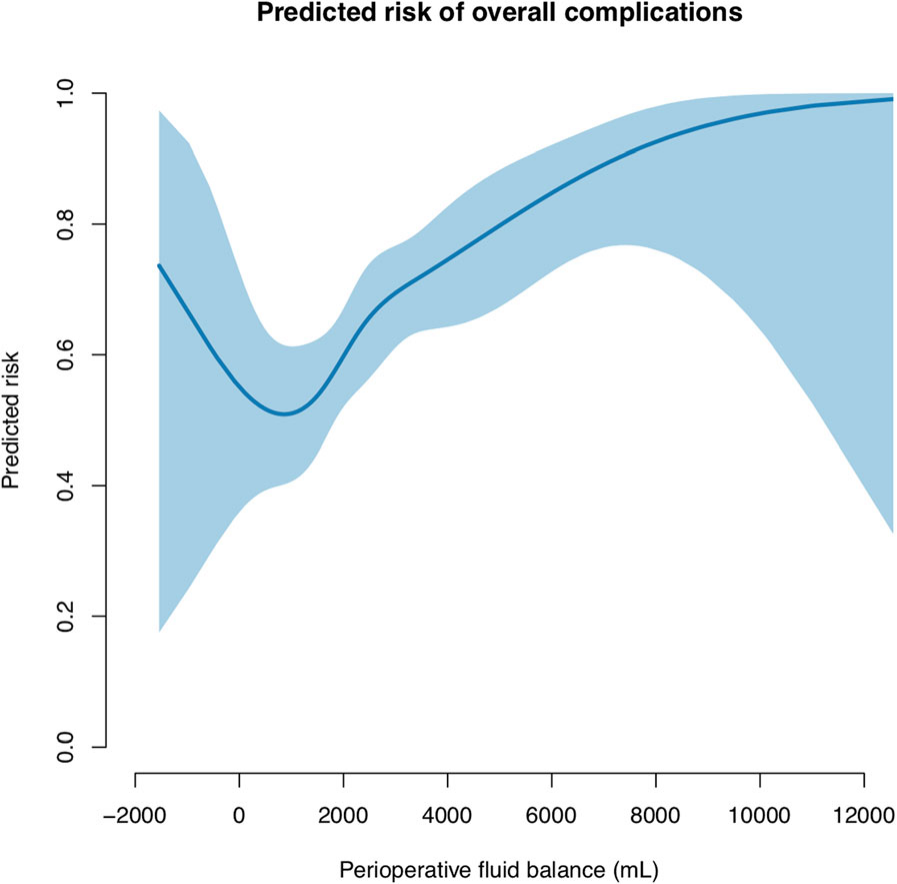
The predicted risk of overall complications associated with the perioperative fluid balance following emergency gastrointestinal surgery. The blue line shows the predicted risk of a complication. The shaded area is the 95% confidence interval. We used a generalized additive model with smoothing splines and four degrees of freedom. The parametric effect is *p* < 0.001 and the non-parametric effect is *p* = 0.572. The parametric calculation tests whether the fluid balance is linear associated with complications. The non-parametric analysis tests whether smoothing splines adds further precision to a linear relation of the model. A *p*-value < 0.01 is considered significant

**Fig. 2:**
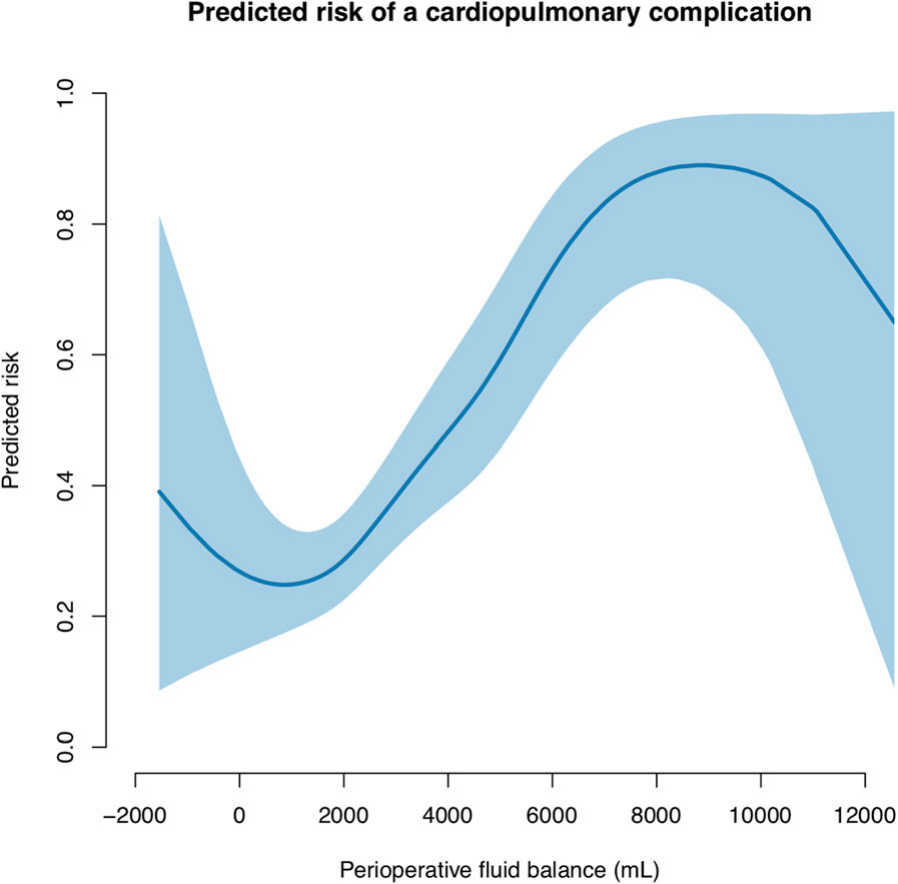
The predicted risk of a cardiopulmonary complication associated with the perioperative fluid balance following emergency gastrointestinal surgery. The blue line shows the predicted risk of a complication. The shaded area is the 95% confidence interval. We used a generalized additive model with smoothing splines and four degrees of freedom. The parametric effect is *p* < 0.001 and the non-parametric effect is *p* = 0.015. The parametric calculation tests whether the fluid balance is linear associated with complications. The non-parametric analysis tests whether smoothing splines adds further precision to a linear relation of the model. A *p*-value < 0.01 is considered significant

**Fig. 3:**
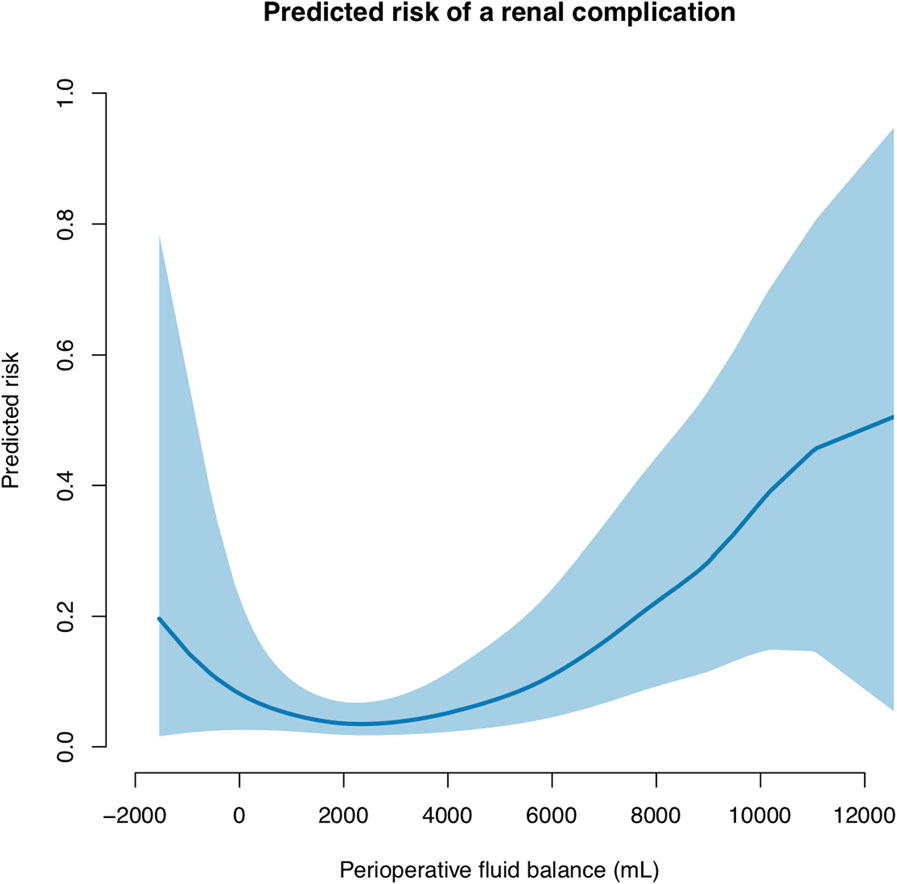
The predicted risk of a renal complication associated with the perioperative fluid balance following emergency gastrointestinal surgery. The blue line shows the predicted risk of a complication. The shaded area is the 95% confidence interval. We used a generalized additive model with smoothing splines and four degrees of freedom. The parametric effect is *p* < 0.001 and the non-parametric effect is *p* = 0.080. The parametric calculation tests whether the fluid balance is linear associated with complications. The non-parametric analysis tests whether smoothing splines adds further precision to a linear relation of the model. A *p*-value < 0.01 is considered significant

The association between the predicted risk of complications and the perioperative fluid balance on a continuous scale is presented in Figs. 1, 2, and 3 and Supplementary Figs. S1 and S2. The figures show that an increased perioperative fluid balance is associated with an increased risk of overall, cardiopulmonary, renal, infectious, or wound related complications. A U-shaped association between the perioperative fluid balance and the predicted risk of cardiopulmonary or renal complications is a good fit. The predicted risk of a cardiopulmonary complication is at a minimum at a perioperative fluid balance approximating 0–2 L, whereas the minimal risk of renal complications is at a fluid balance approximating 1.5–3.5 L.

### Major complications and death

A total of 111 (32.5%) patients developed a major complication (CDC ≥ 3). The risk of a major complication was not significantly associated with the liberal group (OR 1.6 (95% CI 1.0 to 2.7), *p* = 0.077) (Table 3). However, the association between the predicted risk of a major complications and the perioperative fluid balance on a continuous scale showed a U-shaped relation suggesting an optimal fluid balance of approximately 1–3 L (Supplementary Fig. S3). The overall risk of death was 25.4%. The risk of death was not associated with the perioperative fluid balance.

### Sensitivity analysis

We analyzed our data after excluding the 51 patients with a preoperative sepsis score of 3–4 and three patients of which data were missing. The risk of complications remained largely unchanged (Supplementary Table S1). Likewise, analyzing the data without the 68 patients admitted to the ICU immediately after surgery did not change the risk of complications (Supplementary Table 2). Of the patients admitted directly to the ICU after surgery, 31 had a preoperative sepsis score of 3–4 and 29 had postoperative hypotensive episodes of which 24 belonged to the liberal fluid group.

## Discussion

Our study of patients undergoing emergency surgery for gastrointestinal obstruction or perforation showed a perioperative fluid balance of 3.6 L [IQR 3.0 to 5.3] compared with 1.6 L [IQR 1.0 to 2.0] to be significantly associated with a higher risk of postoperative complications, especially cardiopulmonary complications. The correlation remained robust after the exclusion of patients with preoperative severe sepsis or patients directly admitted at the ICU following surgery. The predicted risk of cardiopulmonary and major complications were at a minimum at a perioperative fluid balance of 0–2 L, whereas the predicted risk of renal complications were at a minimum at a fluid balance of 1.5–3.5 L.

A little is known about the influence of the perioperative fluid therapy on postoperative complications in patients undergoing emergency gastrointestinal surgery. One pilot study randomized 29 patients undergoing emergency abdominal surgery to two different fluid strategies (Harten et al., 2008). The perioperative fluid balance was 2.1 L vs 2.9 L. No difference in renal function was found. In an early terminated study, 50 patients with severe sepsis undergoing mixed emergency surgery were randomized to two different goal directed fluid strategies (Pavlovic et al., 2016). The crystalloid administration was 5.6 L vs 5.9 L, and a significant increase in cardiac complications was found in the “liberal” group, most likely due to the protocoled dobutamine administration. A recent randomized trial compared a pressure-guided (standard) with a flow-guided (goal-directed) fluid strategy in major emergency gastrointestinal surgery. The fluid volumes given on the day of surgery was 3984 vs. 3130 ml respectively. Apart from a longer hospital stay in the flow group, no difference in outcome between the groups was found (Aaen et al., 2021).

We divided the patients into two groups, a liberal and a restrictive, after the intravenous fluid volume given; however, the as discussed below, the terms are not well defined in the literature.

We found more cardiopulmonary complications in the patients given a liberal fluid therapy. The group also received more vasopressors postoperatively. The dominating drug given was norepinephrine, which for most parts was given in the intensive care unit. Even so, our result remained robust in the sensitivity analysis when excluding patients directly admitted to the intensive care unit. This indicates that cardiopulmonary complications are not related to the greater use of vasoactive drugs in the liberal group in our study.

We demonstrated a U-shaped correlation between the fluid balance and postoperative complications. This has previously been suggested in meta-analysis of studies comparing restrictive vs. liberal fluid strategies during elective abdominal surgery (Bundgaard-Nielsen et al., 2009; Varadhan & Lobo, 2010). Some studies show a positive result from a restrictive perioperative fluid strategy (Nisanevich et al., 2005; Lobo et al., 2002; Brandstrup et al., 2003) while others report no effect or even a negative effect of a restrictive perioperative fluid strategy (MacKay et al., 2006; Kabon et al., 2005; Holte et al., 2007). The varying results may relate to the circumstance that a restrictive perioperative fluid strategy in one study might resemble a liberal fluid strategy in another study and that different groups of complications are used as outcome (Kabon et al., 2005; Kalyan et al., 2013). In emergency surgery, no method exists to define fluid balance, and the patients are not in balance when arriving to the hospital. Central hemodynamic parameters to measure fluid responsiveness have been proposed as indicators for normovolemia, but superiority to this approach has not been shown.

Our results suggest that the risk of cardiopulmonary and renal complications is differently associated with the perioperative fluid balance. Findings were in agreement with a registry study of patients admitted for elective non-cardiac surgery. Shin and colleagues included 92,000 patients in the study and divided the group in quintiles according to the fluid administration. They found a perioperative fluid administration of > 2.7 L to be significantly associated with an increased risk of respiratory complications, acute kidney injury, and mortality at 30 days (Shin et al., 2017). Additionally, a too restrictive perioperative fluid administration of ≤0.9 L was associated with an increased risk of acute kidney injury, thus suggesting a U-shaped correlation between the fluid administration and the incidence of complications. The study implies a more beneficial outcome in the group of patients receiving a perioperative fluid infusion of 6–7 mL kg^−1^ h^−1^. In similarity, we found a more favorable outcome of a perioperative fluid balance of 1.6 L comparable to a fluid administration of 5.9 mL kg^−1^ h^−1^ for overall and cardiopulmonary complications. Our data suggest that renal function might benefit from a greater fluid administration, and are supported by the study including the largest number of elective surgical patients randomized to a liberal versus restricted fluid strategy: more patients with renal failure were found in the restricted group. Noteworthy, the protocol for that trial did not recommend fluid administration to patients with postoperative oliguria (Myles et al., 2017).

The limitations of our study lay within the retrospective design. The baseline data suggest a possible bias by indication: more patients in the liberal group had gastrointestinal perforation with sepsis and a high ASA score. We chose to adjust for the ASA score. Severe sepsis and co-morbidities are both inherent in the ASA score and as such dependent variables. In addition, more patients in the liberal group had hypotensive episodes treated with IV-fluid and/or vasopressors. We accommodated this by adjusting for the use of vasopressors in the regression model. However, we did not distinguish between different vasoactive drugs, nor a single- versus continuous administration. Blood loss, hypotension, and sepsis are likely to prompt fluid administration but are also linked with increase in morbidity which challenge interpretation of study results (Vincent et al., 2002; Abbott et al., 2018; Mouncey et al., 2015b). However, the sensitivity analysis excluding the patients with preoperative severe sepsis did not change the result, and the difference in blood loss between the groups was minimal (Table 2). We did not register and include the anesthesia used in our analysis (McLean et al., 2015). The anesthetists from the participating hospitals use for most parts propofol, remifentanil, and if indicated rocuronium. Our fluid data relied on the intra- and immediate postoperative period, but not the preoperative or later postoperative period. This is in accordance with most studies in the field.

The strengths of our study are the detailed prospectively registered record-data of perioperative fluid administration. Our data included fluid given as iv-medicine which is often omitted in other studies. Further, double registration of the fluid data and complications was performed to ensure the completeness of available data and avoid misclassification of complications. We adjusted for known confounders influencing the fluid administration and the postoperative complications, further strengthening our findings. The multicenter design strengthens external validity of the study results. Yet, the design has inherent limitations and causal relations are for future trials to explore.

## Conclusion

With reservations to the inherent limitations in the study design, we found a perioperative fluid balance above 2.5 L to be significantly associated with an increased risk of overall and cardiopulmonary complications following emergency surgery for gastrointestinal obstruction or perforation. The predicted risk of complications demonstrates a U-shaped correlation with the perioperative fluid balance. A perioperative fluid balance of 0–2 L was associated with the fewest cardiopulmonary complications. The equivalent estimate was 1.5–3.5 L for renal complications. Our findings support our thesis that avoiding fluid overload in patients undergoing emergency gastrointestinal surgery may reduce the risk of complications.

## Supplementary information

**Additional file 1:.** Supplementary Fig. S1.

**Additional file 2:.** Supplementary Fig. S2.

**Additional file 3:.** Supplementary Fig. S3.

**Additional file 4:.** Supplementary Table S1.

**Additional file 5:.** Supplementary Table S2.

## Data Availability

Please contact the author for data requests.

## Abbreviations

ASA: American Society of Anesthesiologists’ physical status classification
CDC: Clavien-Dindo classification
CI: Confidence interval
ICU: Intensive care unit
IQR: Interquartile range
OR: Odds ratio
