# Supplementary material for: Perioperative fluid administration and complications in emergency gastrointestinal surgery—an observational study
Source: Perioper Med (Lond). 2022 Feb 22;11:9. doi: 10.1186/s13741-021-00235-y (PMC8862386; doi:10.1186/s13741-021-00235-y)
Supplement: Supplementary file 1 — Additional file 1:. Supplementary Fig. S1. [file 13741_2021_235_MOESM1_ESM.docx]

Supplementary Figure S1.

Predicted risk of an infectious complication associated with the peri-operative fluid balance following emergency gastrointestinal surgery

The blue line shows the predicted risk of a complication. The shaded area is the 95% confidence interval. We used a generalised additive model with smoothing splines and four degrees of freedom. The parametric effect p=0.004. The non-parametric effect p=0.358. The parametric calculation tests whether the fluid balance is linear associated with complications. The non-parametric analysis tests whether smoothing splines adds further precision to a linear relation of the model. A p-value <0.01 is considered significant.
