# Supplementary material for: Perioperative fluid administration and complications in emergency gastrointestinal surgery—an observational study
Source: Perioper Med (Lond). 2022 Feb 22;11:9. doi: 10.1186/s13741-021-00235-y (PMC8862386; doi:10.1186/s13741-021-00235-y)
Supplement: Supplementary file 4 — Additional file 4:. Supplementary Table S1. [file 13741_2021_235_MOESM4_ESM.docx]

Supplementary Table S1.

Logistic regression analysis on the association between a peri-operative fluid balance and post-operative complications following emergency gastrointestinal surgery

– only patients with a pre-operative sepsis-2 score of 0-2

|  | Conservative group  (fluid balance ≤2.5L),  n = 162 | Liberal group  (fluid balance >2.5L),  n = 126 | | Crude | |  | Adjusted analysis ¤ | |
| --- | --- | --- | --- | --- | --- | --- | --- | --- |
|  | No. of patients (%) | No. of patients (%) | | OR* (CI95%) | p |  | OR (CI95%) | p |
| Overall complications | 86 (53) | 96 (76) |  | 2.8 (1.7-4.8) | <0.001 |  | 2.7 (1.5-4.8) | <0.001 |
| Wound-related | 36 (22) | 42 (33) |  | 1.8 (1.0-3.0) | 0.036 |  | 1.8 (1.0-3.3) | 0.058 |
| Cardiopulmonary | 36 (22) | 60 (48) |  | 3.2 (1.9-5.3) | <0.001 |  | 2.8 (1.5-5.3) | <0.001 |
| Renal | 5 (3) | 9 (7) |  | 2.4 (0.8-8.0) | 0.122 |  | - | - |
| Infectious | 63 (39) | 69 (55) |  | 1.9 (1.2-3.1) | 0.008 |  | 1.7 (1.0-2.8) | 0.059 |

¤) Clinical risk factors adjusted for in the model: Sex, age in the potency, ASA class (dichotomised at ASA class 3), use of epidural analgesia (yes or no), use of vasopressors (yes or no), the type of surgery (bowel resection, palliative surgery or other procedures), gastrointestinal obstruction or perforation, and the Hospital (Holbæk, Slagelse, or Køge). *) OR: Odds ratio, 95% CI: 95% confidence interval. A p-value <0.01 is considered significant.
